# Prediction of Functional Sites Based on the Fuzzy Oil Drop Model

**DOI:** 10.1371/journal.pcbi.0030094

**Published:** 2007-05-25

**Authors:** Michał Bryliński, Katarzyna Prymula, Wiktor Jurkowski, Marek Kochańczyk, Ewa Stawowczyk, Leszek Konieczny, Irena Roterman

**Affiliations:** 1 Department of Bioinformatics and Telemedicine, Jagiellonian University–Collegium Medicum, Kraków, Poland; 2 Faculty of Chemistry, Jagiellonian University, Kraków, Poland; 3 Faculty of Physics, Astronomy and Applied Computer Science, Jagiellonian University, Kraków, Poland; 4 Institute of Medical Biochemistry, Jagiellonian University–Collegium Medicum, Kraków, Poland; University of California San Diego, United States of America

## Abstract

A description of many biological processes requires knowledge of the 3-D structure of proteins and, in particular, the defined active site responsible for biological function. Many proteins, the genes of which have been identified as the result of human genome sequencing, and which were synthesized experimentally, await identification of their biological activity. Currently used methods do not always yield satisfactory results, and new algorithms need to be developed to recognize the localization of active sites in proteins. This paper describes a computational model that can be used to identify potential areas that are able to interact with other molecules (ligands, substrates, inhibitors, etc.). The model for active site recognition is based on the analysis of hydrophobicity distribution in protein molecules. It is shown, based on the analyses of proteins with known biological activity and of proteins of unknown function, that the region of significantly irregular hydrophobicity distribution in proteins appears to be function related.

## Author Summary

We present here a method of defining functional site recognition in proteins. The active site (enzymatic cavity or ligand-binding site) is localized on the basis of hydrophobicity deficiency, which is understood as the difference between empirical (dependent on amino acid positions) and idealized (3-D Gauss function, or Fuzzy Oil Drop model) distribution of hydrophobicity. It is assumed that the localization of amino acids representing a high difference of hydrophobic density reveals the functional site. The analysis of the structure of 33 proteins of known biological activity and of 33 proteins of unknown function (with comparable polypeptide chain lengths) seems to verify the applicability of the method to binding cavity localization. The comparative analysis with other methods oriented on biological function is also presented. The validation of predictability accuracy is shown with respect to the enzyme classes.

## Introduction

Because of the growing number of structural genomics projects oriented toward obtaining a large number of protein structures in rapid and automated processes [1–4], there is a need to predict protein function (or its functionally important residues) by examining its structure. There have been a variety of efforts in this direction. Some of the techniques used to identify functionally important residues from sequence or structure are based on searching for homologue proteins of known functions [5–8]. However, homologues, particularly when the sequence identity is below 25%, need not have related activities [9–11]. Geometry-based methods have shown that the location of active site residues can be identified by searching for cavities in the protein structure [12] or by docking small molecules onto the structure [13]. The cave localization in silico has been presented on the basis of the characteristics of the normal created for each surface piece [14]. The complex analysis of protein interfaces and their characteristics versus highly divergent areas is presented by Jimenez [15]. Several experimental studies have shown that mutation of residues involved in forming interfaces with other proteins or ligands can also be replaced to produce more stable, but inactive proteins [16–19]. On this basis, several effective algorithms were developed [20,21]. Finally, structural analysis coupled with measures of surface hydrophobicity have been used to identify sites on the surfaces of proteins involved in protein–protein interactions [22].

The Fuzzy Oil Drop (FOD) model presented in this paper is based on an external hydrophobic force field [23–27]. The role of hydrophobic interactions in protein folding [28–31] as well as in protein structure stabilization [32–36] has been known since the classic oil drop model of representing the hydrophobic core in proteins was introduced by Kauzmann [37]. According to this model, the hydrophobic residues tend to be placed in the central part of the protein molecule and in hydrophilic residues on the protein's surface [38–40]. Even the recognition of native versus nonnative protein structures can be to some extent differentiated on the basis of spatial distribution of amino acid hydrophobicity [41–43]. The importance of hydrophobicity distribution has been emphasized, particularly for Rosetta development, when the description of the hydrophobic core significantly increased the performance of the Rosetta program [44]. The discrete system of ellipsoidal centroids was introduced to estimate the concentration of hydrophobic residues, in particular protein zones [44]. The nonrandom hydrophobicity distribution has been proven by Irbäck et al. [45]. However, it was suggested that the core region is not well described by a spheroid of buried residues surrounded by surface residues due to hydrophobic channels that permeate the molecule [46,47]. The FOD model was initially used to simulate the hydrophobic collapse of partially folded proteins. Those structural forms were assumed to represent the early stages of folding (in silico); that model is presented elsewhere [48–50]. The comparison of structures received by folding simulations with their native forms revealed, however, some unexpected results. In the case of native structures, the idealized hydrophobicity distribution satisfying the oil drop–like hydrophobicity partitioning compared with the empirically observed hydrophobicity differs in a specific manner. The high discrepancies between observed and theoretical hydrophobicities within FOD are observed in the area of the binding site [23–26]. It can even be generalized that the location of hydrophobicity differences seems to represent an aim-oriented discrepancy. This simple observation gave us the opportunity to develop a method that was able to recognize functional sites or residues in a protein structure.

In this study, the FOD model is applied to 33 proteins of known function and 33 proteins of unknown function that resulted from structural genomics projects.

## Materials and Methods

### Data

The 33 proteins of known biological activity (Table 1) were selected to verify the reliability of the method. Most of these proteins are enzymes that have well-defined biological function and are deposited in the Catalytic Site Atlas (http://www.ebi.ac.uk/thornton-srv/databases/CSA), a database of templates representing different catalytic mechanisms [51]. The residues identified in this database as active site were used as the criteria to verify the results. Two proteins of known function—rat annexin V, and ButF, the vitamin B_12_-binding protein, which take part in regulation [52] and transport processes [53], respectively—are also included in the test probe.

**Table 1:**
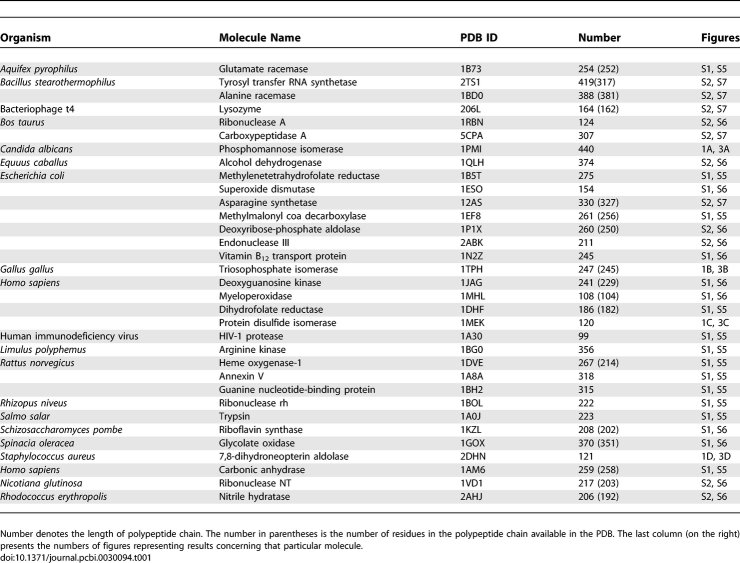
Proteins of Known Function Taken to Analysis

Reports from structural genomics projects [1–4] have described the solution of a number of proteins with unknown functions. The procedure for potential functional site recognition presented in this paper was performed with a set of 33 such proteins deposited in the Protein Data Bank (PDB) (Table 2).

**Table 2:**
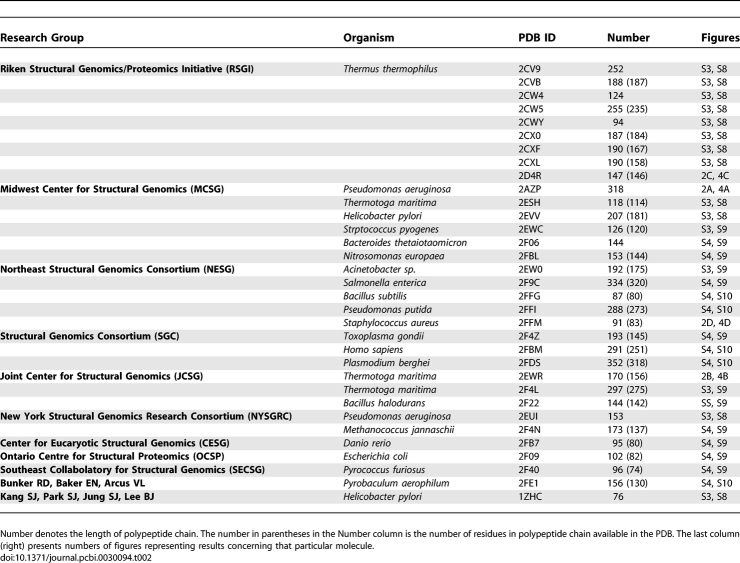
Proteins of Unknown Function Taken to Analysis

The multimeric proteins were represented solely by their first chain in the PDB file. All molecular visualizations were created with Pymol software [54].

### Hydrophobic Force Field

The FOD hydrophobic force field is based on the assumption that the theoretical hydrophobicity distribution in proteins is represented by the 3-D Gaussian function. The value of this function in a particular *j*-th point within the space occupied by a protein represents the hydrophobicity density at this point:

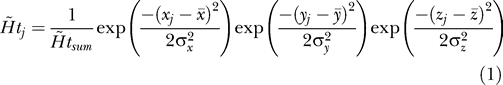



Where 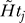 is the theoretical (expected) hydrophobicity of the *j*-th point, *σ_x_*, *σ_y_*, *σ_z_* are the standard deviations, which depend on the length of polypeptide under consideration [23–26] and the point 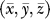 is localized in the center of coordinate system (0,0,0) of the highest theoretical hydrophobicity. This simplifies Equation 1:

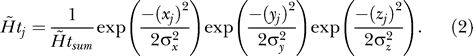
The molecule is oriented according to the following procedure: the longest distance between two effective atoms determines the *z*-axis, and the longest distance between projections on the *x*–*y* plane determines the *x*-axis.

For this orientation of molecules in the coordinate system, the values of *σ_x_*, *σ_y_*, *σ_z_* parameters are calculated as one-third of the highest *x*, *y*, or *z* coordinates of the effective atom increased by 9 Å (cutoff distance for hydrophobic interaction) in each direction. The values of the Gaussian function are standardized to give a value of 1.0.

The second component of this force field is an observed (empirical) hydrophobicity distribution formed by the side chains of a protein molecule, and can be expressed using the original function introduced by Levitt [55]. The *j*-th point collects hydrophobicity 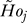 as follows:

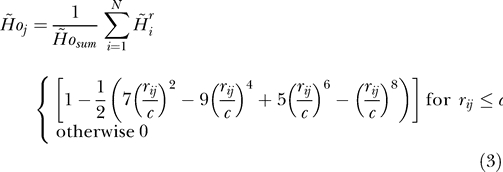
where 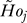 denotes the empirical hydrophobicity value characteristic for the *j*-th point, *N* is the number of residues in a protein, 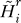 represents the hydrophobicity characteristic for the *i*-th amino acid, *r_ij_* is the distance between the *j*-th point and the geometrical center of the *i*-th residue, and *c* expresses the cutoff distance, which has a fixed value of 9.0 Å, following the original paper [55]. The observed hydrophobicity distribution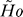 is also standardized. More details concerning the FOD force field are given in recently published papers [23–27].

The similarity of the FOD-based hydrophobic scale with others commonly used for calculations (e.g., the Eisenberg [56] or Doolittle [40] scales) has been shown and discussed in [57]. The differences between these scales seem to be negligible with respect to the problem under consideration. Use of these scales does not change the 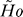 distribution significantly (Equation 3) [57]. The introduction of the FOD-based hydrophobic scale unifies the system for proteins (amino acids) and molecules interacting with proteins, creating stable complexes (ligands).

### 

#### Scoring Function.

Since both theoretical 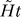 and observed 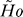 distributions of hydrophobicity are standardized to 1.0 and were calculated for the same set of points (geometrical centers of all residues in a protein), the comparison of these two characteristics is possible. The difference between theoretical and empirical distributions 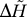 expresses the irregularity of hydrophobic core construction. For the *i*-th residue, 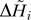 is calculated as follows:

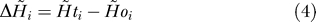
where 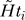 and 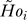 are the theoretical and observed values of hydrophobicity for the geometric center of the *i*-th residue, respectively.

The maxima of 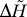 recognize the residues representing the hydrophobicity deficiency, which points out the structural irregularity, usually in a function-related area.

#### Comparative Analysis.

The SuMo and ProFunc methods (both available on the Web, see urls below) were selected to perform the comparative analysis as to functional site recognition.

*SuMo.* SuMo is a Web tool [58] (http://sumo-pbil.ibcp.fr/cgi-bin/sumo-welcome) that predicts the function of proteins based on the chemistry of the bound ligand. Each ligand and macromolecule part is divided into sets of arbitrary predefined chemical groups. The active site is recognized by a comparison of a minimum of three chemical groups in both compared molecules. SuMo produces a list of probable active sites on default ranked by the number of SuMo groups involved in each given prediction. The active site is described by a set of amino acids and corresponding chemical groups [59].

*ProFunc.* ProFunc [60] is a Web server (http://www.ebi.ac.uk/thornton-srv/databases/ProFunc) devoted to predicting the function of proteins of known 3-D structure and unknown function. The server provides both sequence- and structure-based methods, which may be used in the analysis of proteins. From the group of structure-based methods available on the server, the “reverse templates” 3-D template–based method [61] was chosen and applied to validate the method presented in this study. According to the reverse-template method, the structure itself is broken up into a large number of templates (each containing three residues) that are scanned against a representative set of structures in the PDB [61]. All the hits obtained are scored and ranked. Other homology/sequence-based tools were not taken into account; only methods of similar (structure-based) methodologies were included.

The coordinates of all protein structures under study were submitted to the server in PDB format. The top reverse template–matching structures of known and unknown functions were used in our comparative analysis.

### Result Verification

The residues annotated in CSA as those playing roles in catalytic activity were used as the gold standard to verify the reliability of the results received according to the FOD model.

To indicate the most meaningful amino acids considered by the FOD model to be located in the functional site, the calculation of percentiles was used to identify the threshold for selection of 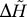 maxima, which are distinguished as belonging to the functional site. It is possible to do so, because the quantitative results expressing the level of 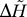 can be taken as the criteria for discrimination. For a set of measurements arranged in order of magnitude, the *p-th* percentile is the value that has *p* percent of the measurements below it and (*100* − *p*) percent above it. In this analysis, the 95th percentile was used. In other words, among the analyzed data, 95% of values were below the 95th percentile threshold, and only the 5% above the threshold was taken into consideration.

The same validation method cannot be used in the SuMo or ProFunc methods because of their different types of output data. They produce only the numbers of amino acids that potentially belong to functional sites and total scores (based on which given set of amino acid residues is assessed and what functional site is proposed). This is why the percentage of commonly classified residues was calculated for each protein molecule by taking the best hit by ProFunc (according to the score value) and the solution most relevant to the FOD-based results by SuMo.

## Results

### Functional Site Recognition in Proteins of Differentiated Biological Activity

The proteins of known biological activity (Table 1) and protein structures of unknown function that resulted from structural genomics projects (Table 2) were examined for the locations of their functional sites. Table 3 summarizes the results of the method application and comparison with experimental observations (CSA classification). The first column presents the protein under consideration and the list of residues recognized by CSA. For two proteins (rat annexin V and ButF), residues that are in direct contact with ligand [62,63] and/or are part of the functional site are given [64].

**Table 3:**
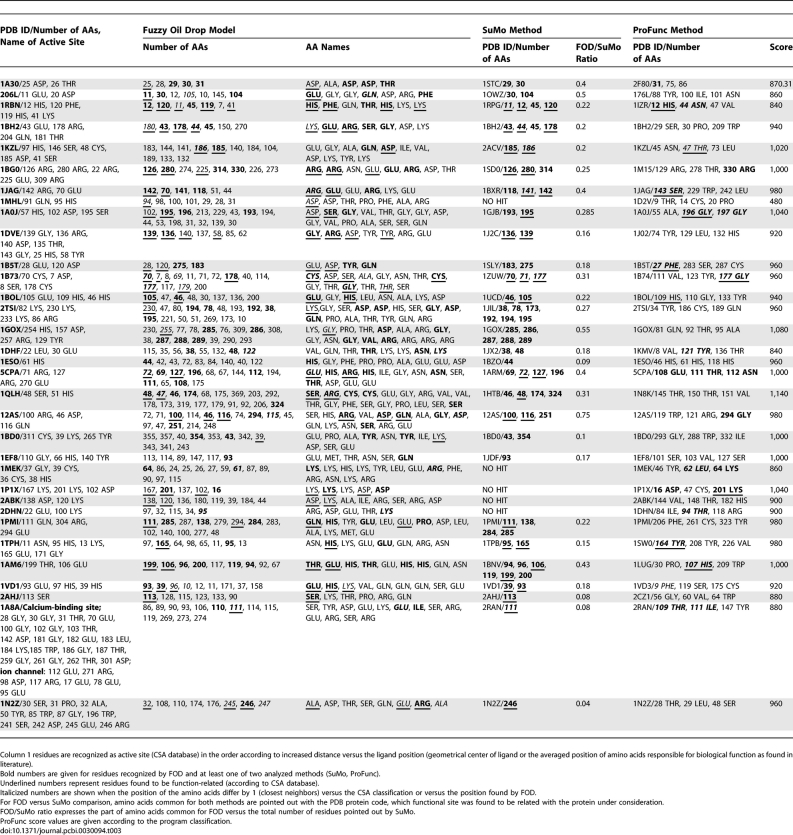
Biological Activity–Related Residues as Recognized in Proteins of Known Biological Function Using Methods Discussed in This Paper

In Table 3, the columns representing FOD results show the numbers of residues recognized by this method: agreement with CSA classification (underlined), and residues defined by two methods—FOD and at least one of two other methods (SuMo, ProFunc) as biological activity-related residues (in bold). Where the position of the amino acids differed by 1 (closest neighbors) versus the CSA classification or versus the position found by SuMo or ProFunc, the numbers are in italics in Table 3. The description of the SuMo and ProFunc columns in Table 3 is given below (Comparative Analysis).

The residues recognized as potentially responsible for binding site creation in proteins of unrecognized biological function are given in Table 4.

**Table 4:**
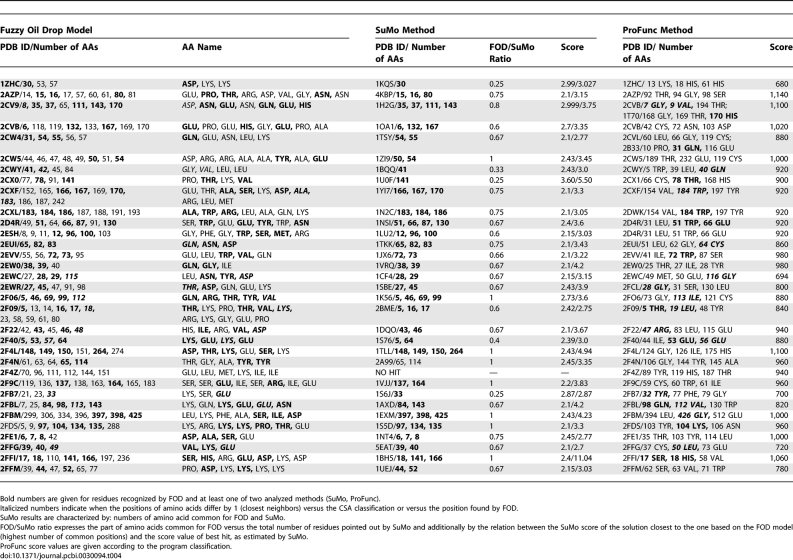
Biological Activity–Related Residues as Recognized in Proteins of Unknown Biological Function Using Methods Discussed in This Paper

**Figure 1:**
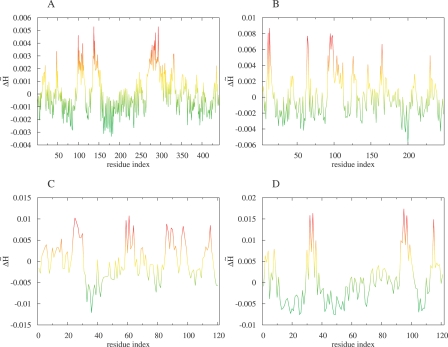
Profile Plots of Hydrophobicity Deviation Δ* H~* per Amino Acid Obtained for Exemplary Proteins of Known Function (A) Phosphomannose isomerase and (B) triosophosphate isomerase are examples of the high agreement with experimental data. (C) Protein disulfide isomerase and (D) 7,8-dihydroneopterin aldolase are examples of low agreement. The common color scale is introduced: red, high Δ* H~*; yellow, middle Δ* H~*; green, low and negative Δ* H~*.

**Figure 2:**
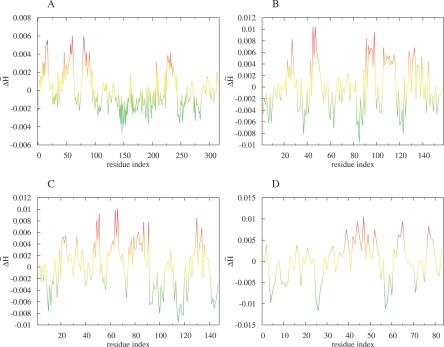
Profile Plots of Hydrophobicity Deviation Δ* H~* per Amino Acid Obtained for Exemplary Proteins of Unknown Function The protein identified in the genome of Pseudomonas aeruginosa (A) and the protein identified in the genome of Thermotoga maritima (B) are examples representing close localization of residues of high Δ* H~*. The protein originated in the Thermus thermophilus genome (C) and the protein originated the Staphylococcus aureus genome (D) are examples of dispersed localization of residues representing high Δ* H~*. The common color scale (same as in Figure 1) is introduced: (low and negative Δ* H~* proteins need additional analysis of their specificity).

Profile plots of 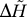 were used to identify the positions recognized by the FOD model as related to functional sites. The profile plots of 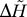 were examined for proteins of known and unknown biological activity (Figures 1, S1, and S2; and Figures 2, S3, and S4; respectively). The residues with the highest 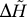 appeared as peaks in the profile plots and were predicted to be functionally important. The values of 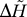 indicate the level of hydrophobicity irregularity. It is interpreted that the higher the 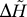 value, the higher the deficiency of hydrophobicity with respect to its idealized distribution according to Gauss function. Thus, the 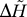 maxima identified as being represented by a particular amino acid point out the residues in the surrounding area where the hydrophobicity deficiency is significant. In most cases, this deficiency is caused simply by the presence of a cavity or by the highly irregular distribution of side chains. The 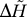 profile together with the color scale visualizes the magnitude of the irregularity. The same scale applied to the 3-D presentation of the protein molecule is able to visualize the location of high 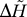 values, particularly in the protein structure. It can be seen that the residues with high 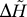 values appear to be placed in close mutual vicinity, often creating a cleft, which can be responsible for ligand (substrate) binding.

The 3-D representations for selected proteins of known function are shown in Figure 3, and for selected proteins of unknown biological function in Figure 4. Other proteins under consideration are presented in Figures S5–S7 and Figures S8–S10.

**Figure 3:**
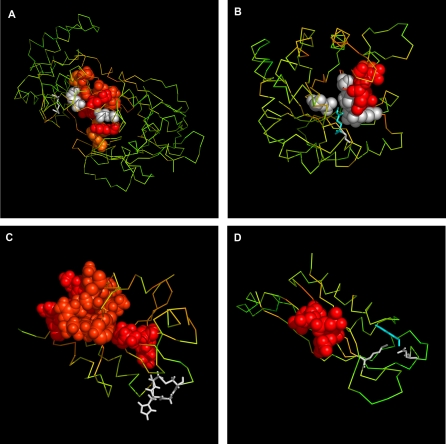
The 3-D Representation of Proteins of Known Biological Activity with Binding Site Recognized Phosphomannose isomerase (A) and triosophosphate isomerase (B) are examples of the high agreement with experimental data. Protein disulfide isomerase (C) and 7,8-dihydroneopterin aldolase (D) are examples of low agreement. Amino acids indicated by FOD as belonging to the binding cavity are in CPK form. The common color scale (same as in Figure 1) is introduced. The white color denotes the experimentally verified amino acids as active site (identification according to the CSA database).

**Figure 4:**
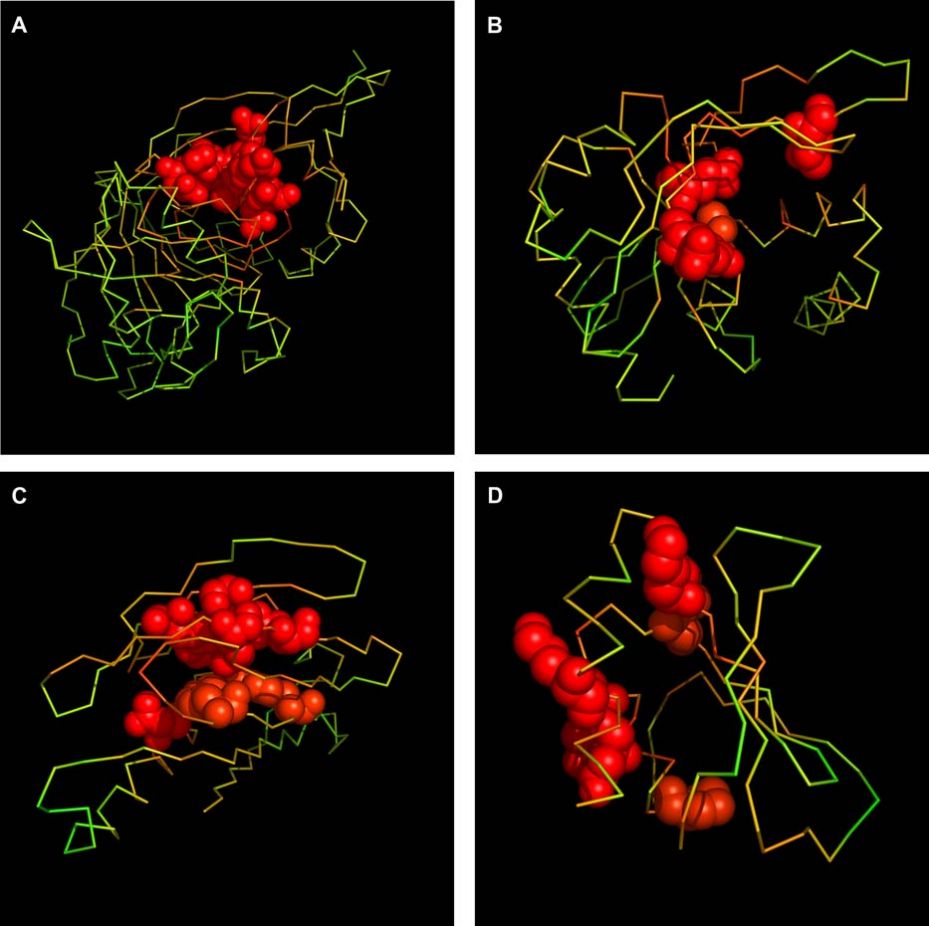
The 3-D Representation of Proteins of Unknown Biological Activity with Binding Site Recognized The protein identified in the Pseudomonas aeruginosa genome (A) and the protein identified in the Thermotoga maritima genome (B) are examples representing close localization of residues of high Δ* H~*. The protein originated in the Thermus thermophilus genome (C) and the protein originated in the Staphylococcus aureus genome (D) are examples of dispersed localization of residues representing high Δ* H~*. The common color scale (same as in Figure 1) is introduced.

The color scale expressing the magnitude of 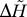 is as follows: red, high 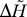 ; yellow, average 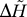 ; green, low and negative 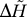 . The white color denotes the experimentally verified amino acids as responsible for catalytic activity (according to the CSA database). In most cases, the set of amino acids selected according to the FOD model is larger than the set of residues classified by CSA. This is because the 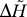 profile also selects amino acids that are close in space, which create well-defined putative cavities that accompany the residues responsible for enzymatic activity. Amino acids indicated by FOD as belonging to the binding cavity are in space filling form.

The molecules presented in Figure 3A and 3B are selected to show the best results; the molecules presented in Figure 3C and 3D demonstrate the cases of low accordance. Some of the protein molecules with high 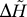 values shown in Figure 3A and 3B appeared to be highly accordant to the active site location. Other proteins with high 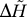 values (Figure 3C and 3D) are not exactly located in the positions of the amino acids that make up the catalytic site. Nevertheless, the analysis of the larger set of proteins may suggest that the specificity of the mutual location of the residues represented by high 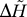 values versus the position of the enzymatic site may be classified according to enzyme specificity.

One hypothesis is that the residues responsible for complex fixation (protein and ligand or substrate) were selected by the FOD model. Another explanation for the mismatch between experimentally identified and automatically identified residues is simply that for multimeric chains, only the first chain was present in the analysis.

### Comparative Analysis

The results summarize the comparison of the model applied to identify the ligand-binding site and two other methods dedicated to the same purpose: ProFunc and SuMo are given in Table 3 for proteins of known biological function and in Table 4 for proteins of unknown biological function. Table 3 presents the list of proteins (the PDB accession numbers are given) accompanied by the amino acids identified as function-related according to CSA classification.

SuMo results (for each SuMo search in question) show the comparison with the FOD model for only one example of a functional site found by SuMo and present the residue numbers, which appeared to be common for these two methods (column 4 of Table 3). The limitation to compare only one SuMo result for one search is caused by the specificity of output generated by the SuMo procedure, which produces an enormous number of possible solutions for one particular protein molecule (in most cases, thousands of variants). Each solution is presented with regard to another protein (PDB number given), the functional site of which seems to be related to that found in the molecule under analysis. This procedure proposes a list of functional sites that sometimes represent changed functionality (e.g., ligands of different structure/characteristics are bound). One functional site with a functional site of the same/closest properties is selected. The presentation of all results is impossible to present here in complete form.

In column 5 of Table 3, the ratio of commonly recognized residues to the number of all residues recognized by SuMo for that hit is shown. As we see, the total number of amino acids classified by SuMo in most cases is the same or exceeds the number identified by the FOD model.

The numbers given in the last two columns (ProFunc) of Table 3 represent positions of amino acids recognized by ProFunc by its best hit and method score. This is why the number of commonly recognized residues (given in bold) is lower than in the SuMo comparison.

The results describing the analysis of proteins of unknown biological function are shown in Table 4. The presentation is similar to that for proteins of known biological function with an obvious lack of underlined positions (no CSA classification available). The SuMo results are additionally characterized by the relation between the SuMo score of the solution closest to that based on the FOD model (highest number of common positions) and the score value of best hit, as estimated by SuMo.

The comparison of the methods selected for analysis is generally very difficult. The SuMo and ProFunc methods represent the methodology of the stochastic nature. The FOD seems to be a more heuristic method. SuMo and ProFunc produce very large outputs with long lists of possible approaches. Each of them is characterized by the scoring number calculated according to the number of contacts (pairs of amino acids) responsible for ligand–protein interaction. However, the number of residues commonly recognized by at least two analyzed methods seems to be quite high.

Taking into account a very large discrepancy in the results of one particular method, the level of mutual accordance seems to be satisfactory.

### Result Validation

Tables 5 and 6 present the results aimed toward validating the FOD model–based results. The values present error levels calculated for the methods under consideration. These calculations take into account the number of mismatched residues versus the CSA, SuMo, and ProFunc classifications. Tables 5 and 6 also include comparisons versus functional site amino acids estimated by the 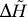 above the 95th percentile value.

**Table 5:**
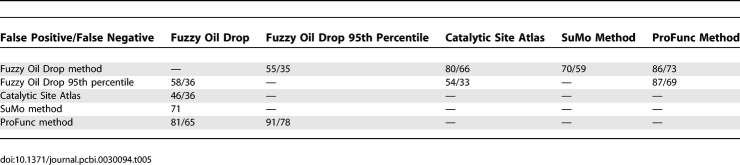
Error Analysis for Proteins of Known Biological Function

**Table 6:**
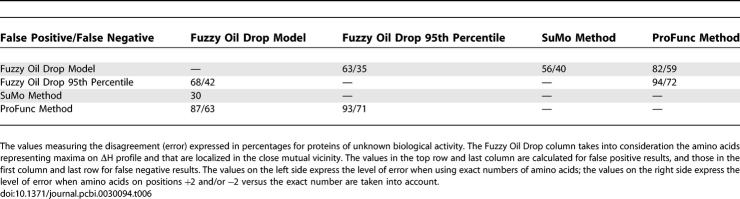
Error Analysis for Proteins of Unknown Biological Function

The proteins of known biological function are characterized in Table 5, and the proteins of unknown biological function are characterized in Table 6. The false negative (below diagonal) and false positive (above diagonal) classifications are given as average (for all analyzed proteins) percentages of mismatched residues.

The comparison is expressed by the level of error measured in the percentage of mismatched residues. The left value in each table cell was calculated by taking into account the exact amino acid numbers. The value on the right side expresses the percentage of mismatched residues when the tolerance of (i + 2)/(i − 2) amino acids (the positions of the residues) is taken into consideration.

The FOD results are based on the 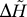 profile along the polypeptide chain. The search for the percentile optimally discriminating the residues belonging to those classified by CSA can be performed. The 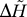 values above the 95th percentile value appeared to be the best approach of local 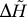 maxima as the criteria for function-related residue classification. The results of the comparison of the 95th percentile are shown in the “FOD 95th percentile” column.

The interpretation of values given in Tables 5 and 6 is as follows. For example, in FOD versus ProFunc cases, 86% of residues found by the FOD method were not selected by ProFunc (false positives). Taking the amino acids with (i + 2)/(i − 2) tolerance, the level decreases to 73%.

In false negative cases, 81% of residues selected by ProFunc were not selected by FOD (65% when closest neighbors were taken into account).

This study is not designed to give a thorough comparison of functional site tools, nor is it meant to review the current advances in this field. Therefore, the mutual comparisons between SuMo and ProFunc, SuMo and CSA, and ProFunc and CSA are not presented here.

Additional analysis summarizing the applicability of the presented method is also shown in Table 7. It is shown that the correctness of the FOD model depends on the enzyme class. Values in Table 7 express the percentages of the residues identified by the FOD method versus those identified by CSA. The highest agreement was found for the EC.3 category (hydrolases), where almost 70% of residues classified by CSA were found by the FOD model. The functional sites in enzymes belonging to the EC.4 (lyases) and EC.6 (ligases) classes were recognized quite well (more than 60%). The lowest agreement was found for the EC.2 class (transferases), where the percentage of correctly predicted amino acids (versus CSA classification) was about 20% (this seems nonrepresentative due to the low number of proteins under consideration in this class).

**Table 7:**
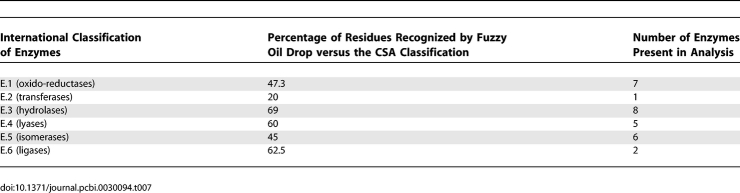
Correctness of the Fuzzy Oil Drop Method as Dependent on the Enzyme Category

The specificity of the active sites in particular enzymatic classes will be analyzed in future publications with respect to the FOD methodology. The larger number of proteins belonging to particular enzyme classes will be taken into consideration in the prospective analysis with respect to the applicability of the FOD model as the tool for functional site recognition. The increased number of proteins representing a particular enzyme class may clarify also the applicability of the method in relation to the detailed type of reaction catalyzed.

## Discussion

The recognition of functional sites in protein molecules is important for the identification of biological activity. The fully automatic method is highly expected. In analogy to the methods applied for protein structure prediction, the ligand-binding site can be recognized on the basis of *comparative methods* (according to CASP [critical assessment of structure prediction] classification). The alternate possibility is to search for a ligand-binding site using *new fold* (according to CASP classification) techniques that use only the structure of individual proteins.

The FOD method presented here identifies the potentially function-related amino acids. In contrast to SuMo and ProFunc, which are based on comparative analysis, the FOD method is of heuristic form, taking as its criterion the individual local hydrophobicity deficiency in a particular protein body.

The ligands' (as cofactors or cosubstrates) presence makes the biological activity possible for some proteins. The enzymatic activity also requires substrate binding. The presence in the cavity of high specificity versus ligand/substrate is needed for this kind of interaction. The location of the cavity (dependent on the protein character) in protein molecules seems to be well recognized by the FOD model.

The part of the protein molecule with high hydrophobic deficiency is recognized as a possible ligand-binding site (or active site). Some results received according to the FOD model seem to be quite satisfactory (Figure 1A and 1B and Figure 3A and 3B). The catalytic mechanisms of enzymes are quite differentiated and require appropriate molecular structures. The analysis of their specificity may clarify the origin of failure (Figure 1C and 1D and Figure 3C and 3D). The possible protein–protein complex creation (not taken into consideration in this analysis) may significantly influence the results (e.g., Figures S1 and S6). Two proteins (in Figures 1C and 3C, and in Figures 1C, 3C, S2, and S7) of common enzymatic specificity (disulphide isomerase) have been recognized on the basis of the FOD method as highly similar with respect to the mutual orientation of residues involved in cavity creation. The specificity of enzymes with respect to their active site construction is the aim of prospective analysis, which will be published soon, as well as analysis of proteins responsible for biological functions other than enzymatic (e.g., proteins responsible for transport as given in Table 3).

The calcium-binding sites in annexin V are not recognized by FOD, although the ion channel–creating residues are pointed out by this method according to expectations for the method of biological function recognition.

The FOD model may also represent the specific hydrophobic environment for protein folding and was initially aimed at the simulation of the hydrophobic collapse of partially folded proteins. The heuristic model of protein folding, according to which the folding polypeptide is directed to follow the hydrophobicity distribution, is represented by the 3-D Gaussian function. The external force field may direct the folding process toward the hydrophobic core creation. The resulting structure appeared to be dissatisfactory, particularly because of the absence of a ligand-binding site in the final structural form. The presence of a ligand in the folding environment may ensure the specific binding cavity creation. Thus, it seems to be important or even necessary.

The comparative analysis of the results of the FOD-based method with the results of SuMo and ProFunc (Tables 3–6) reveals the very high similarity of obtained results. The methods use different criteria for classification. The exhaustive comparative analysis of the results obtained by the application of different methods seems to be necessary and has been taken into consideration; this will be published soon together with explanation of the source of these differences.

The proteins shown in this paper represent mostly enzymes of varying biological activity, the relation of which to the character of the results will be the object of independent research.

It is generally accepted that globular proteins consist of a hydrophobic core and a hydrophilic surface [36,40]. However, the core region is not well described by a spheroid of hydrophobic residues surrounded by hydrophilic residues due to channels that permeate the molecule [46,47]. The FOD model, when applied to protein structure, characterizes the hydrophobicity density in a continuous form by pointing out the irregularities in a hydrophobic core construction disturbing the regularity of hydrophobicity distribution [23–26]. Those irregularities seem to be good markers for ligand-binding sites or functionally important residues.

Methods dedicated to active site recognition have been widely developed: SARIG [65], Q-SITE FINDER [66], HIPPO (SPROUT) [67,68], FEATURE [69–71], THEMATICS [72–74], APROPOS [75], DRUGSITE [76], and LIGSITE [77], to mention just a few. Limitation to two methods (SuMo and ProFunc) for comparative analysis in this paper is due to the very large variability of the models when applied.

The method described in this paper is assumed to be applied for active site identification for a large set of proteins, the structure of which is planned to be generated using different methods (FOD and ROSETTA [78]). The project geared toward biological activity identification in never born proteins (NBPs) is assumed to deliver the molecules of pharmacological application [79,80]. This is the main scientific goal for pharmacology application in the EuChinaGrid project.

The FOD method is available at http://bioinformatics.cm-uj.krakow.pl/activesite.

## Supporting Information

Figure S1 Other Proteins Listed in Table 1 Presented as Described in Figure 1
(5.1 MB TIF)

Figure S2 Continuation of Proteins Listed in Table 1 Presented as Described in Figure 1
(9.6 MB EPS)

Figure S3 Other Proteins Listed in Table 2 Presented as Described in Figure 2
(8.3 MB EPS)

Figure S4 Continuation of Proteins Listed in Table 2 Presented as Described in Figure 2
(7.9 MB EPS)

Figure S5 Other Proteins Listed in Table 2 Presented as Described in Figure 3
(8.0 MB TIF)

Figure S6 Continuation of Other Proteins Listed in Table 2 Presented as Described in Figure 3
(8.0 MB TIF)

Figure S7 Continuation of Other Proteins Listed in Table 2 Presented as Described in Figure 3
(2.9 MB TIF)

Figure S8 Other Proteins Listed in Table 2 Presented as Described in Figure 4
(7.3 MB TIF)

Figure S9 Continuation of Other Proteins Listed in Table 2 Presented as Described in Figure 4
(8.4 MB TIF)

Figure S10 Continuation of Other Proteins Listed in Table 2 Presented as Described in Figure 4
(3.5 MB TIF)

### Accession Numbers

The Protein Data Bank (http://www.rcsb.org/pdb) accession numbers for the proteins discussed in this paper are: rat annexin V (1A8A), ButF (1N2Z), phosphomannose isomerase (1PMI), triosophosphate isomerase (1TPH), protein disulfide isomerase (1MEK), 7,8-dihydroneopterin aldolase (2DHN), protein identified in the Pseudomonas aeruginosa genome (2AZP), protein identified in the Thermotoga maritima genome (2EWR), protein originated in the Thermus thermophilus genome (2D4R), protein originated in the Staphylococcus aureus genome (2FFM), myeloperoxidase (1MHL), and riboflavin synthase (1KZL).

## Abbreviations

CSA: Catalytic Site Atlas
FOD: Fuzzy Oil Drop
