# Supplementary figures and images for: Prediction of Functional Sites Based on the Fuzzy Oil Drop Model
Source: PLoS Comput Biol. 2007 May 25;3(5):e94. doi: 10.1371/journal.pcbi.0030094 (PMC1876487; doi:10.1371/journal.pcbi.0030094)

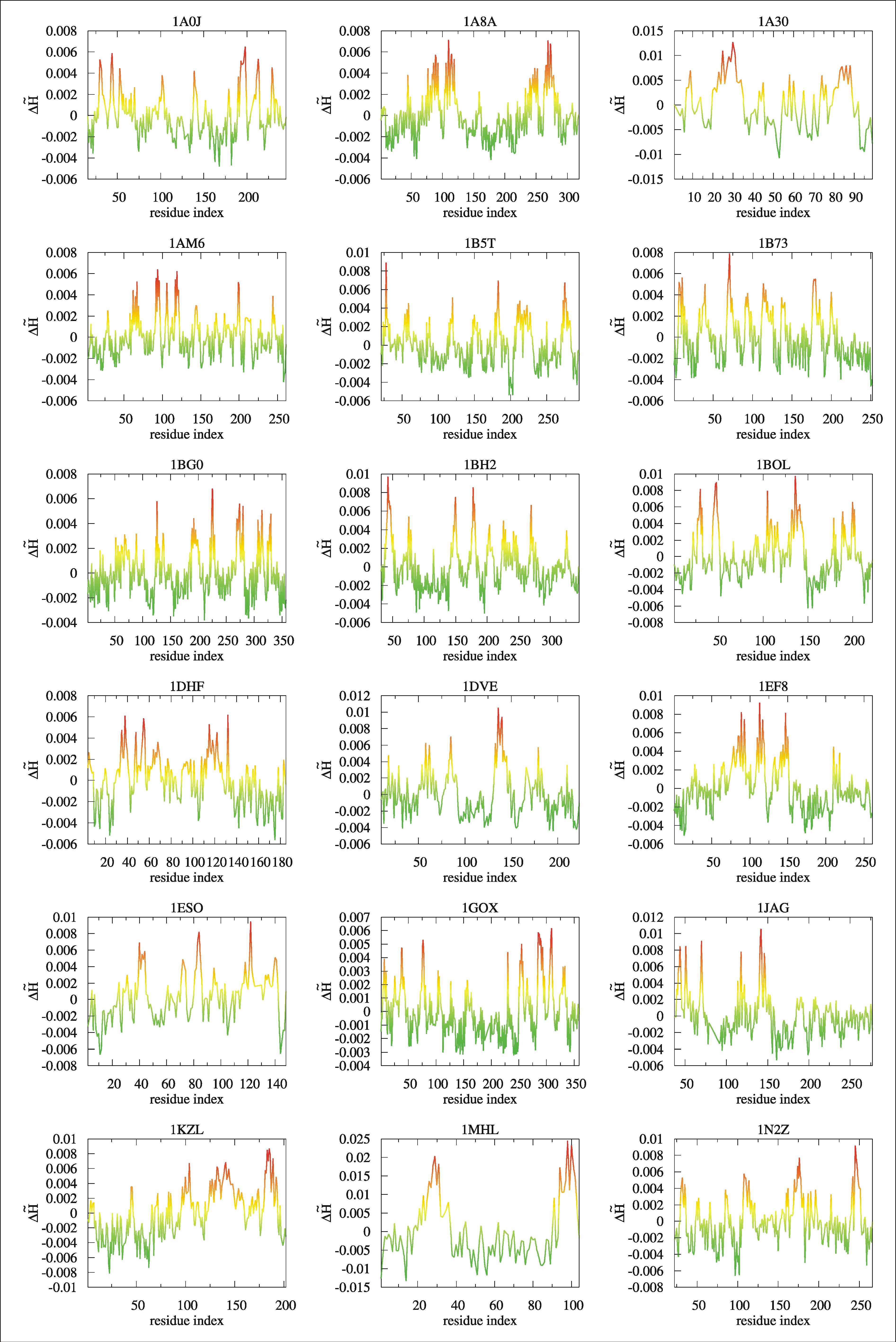

Supplement: Figure S1 — (5.1 MB TIF) [file pcbi.0030094.sg001.tif]

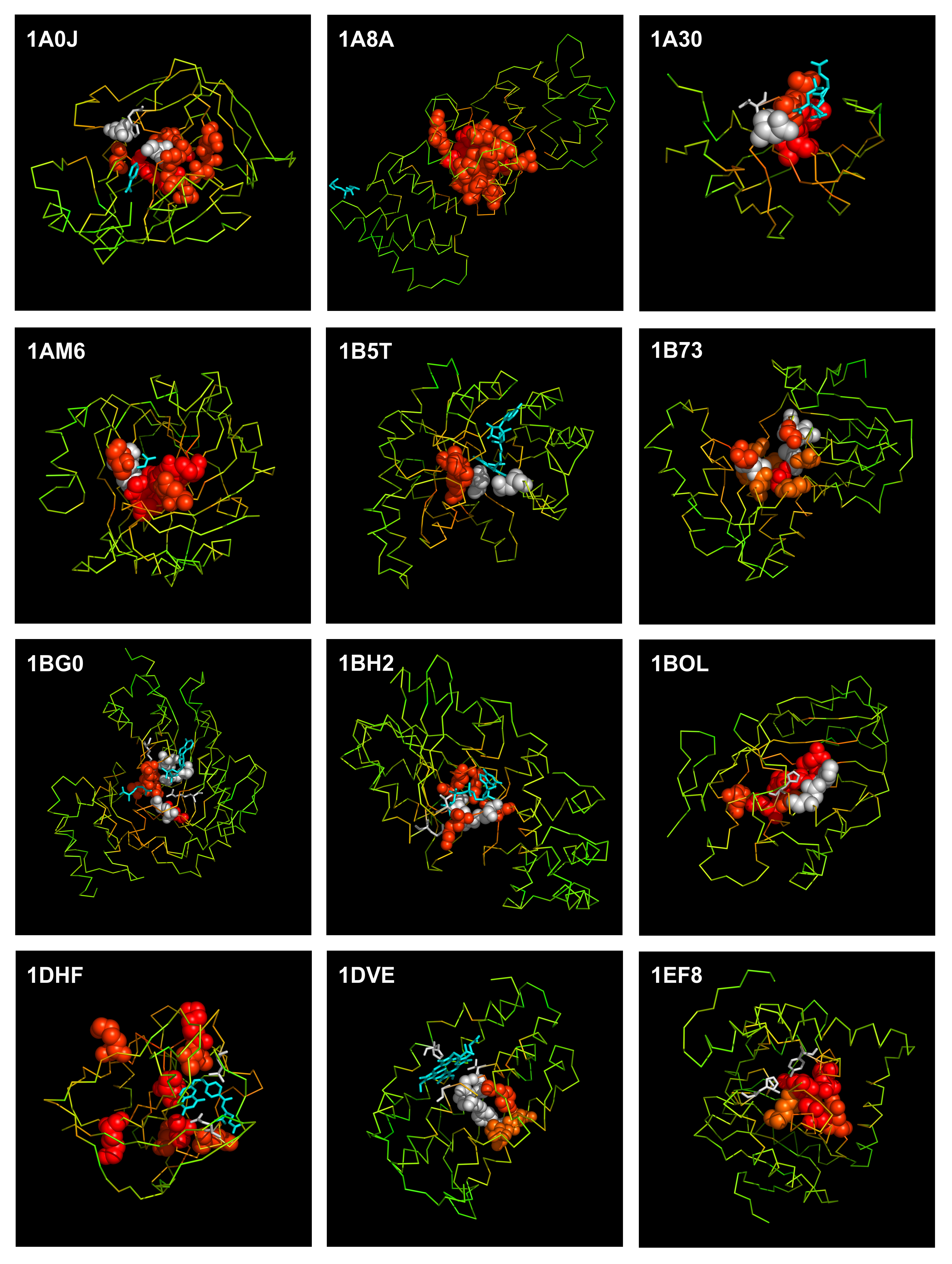

Supplement: Figure S5 — (8.0 MB TIF) [file pcbi.0030094.sg005.tif]

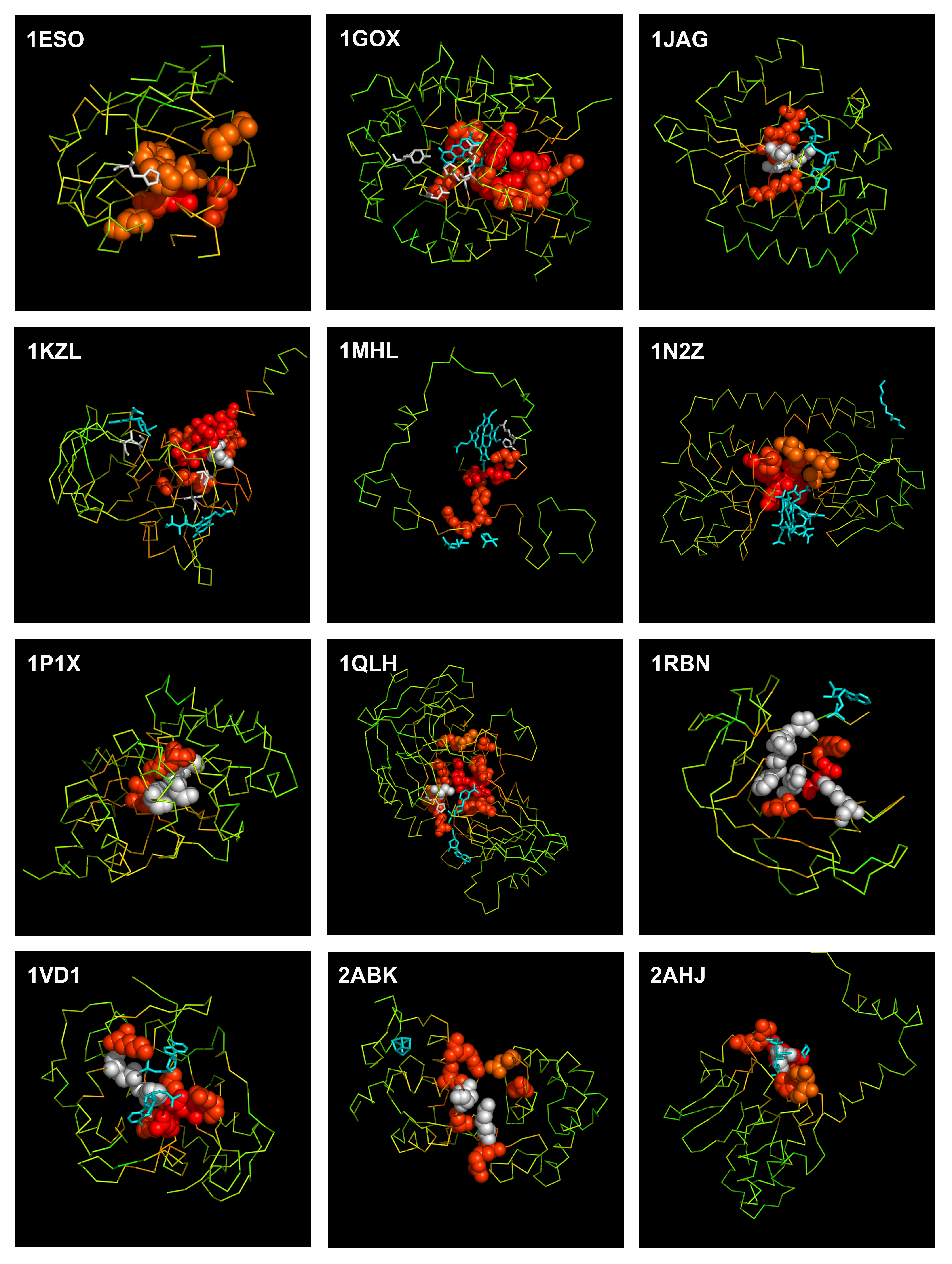

Supplement: Figure S6 — (8.0 MB TIF) [file pcbi.0030094.sg006.tif]

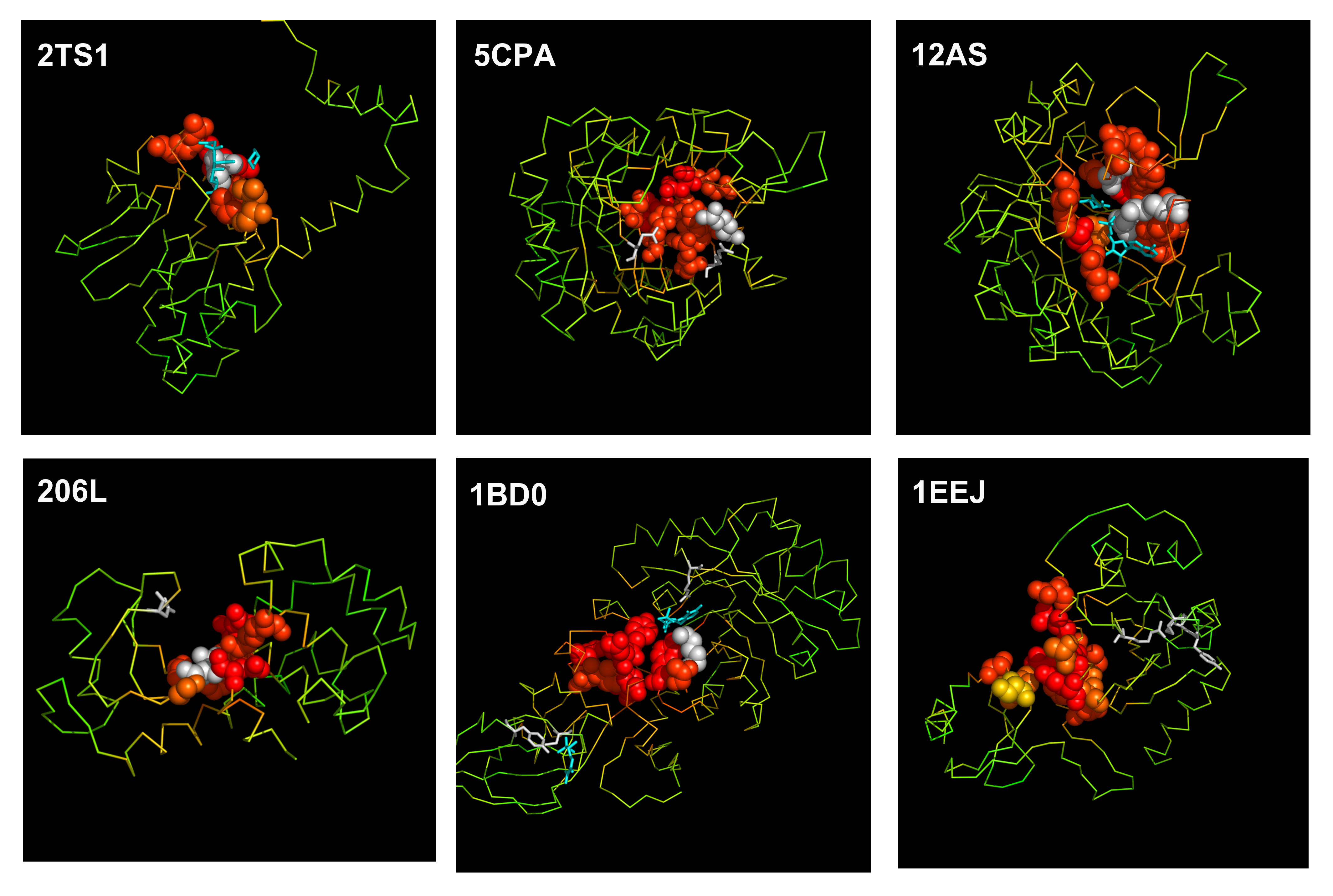

Supplement: Figure S7 — (2.9 MB TIF) [file pcbi.0030094.sg007.tif]

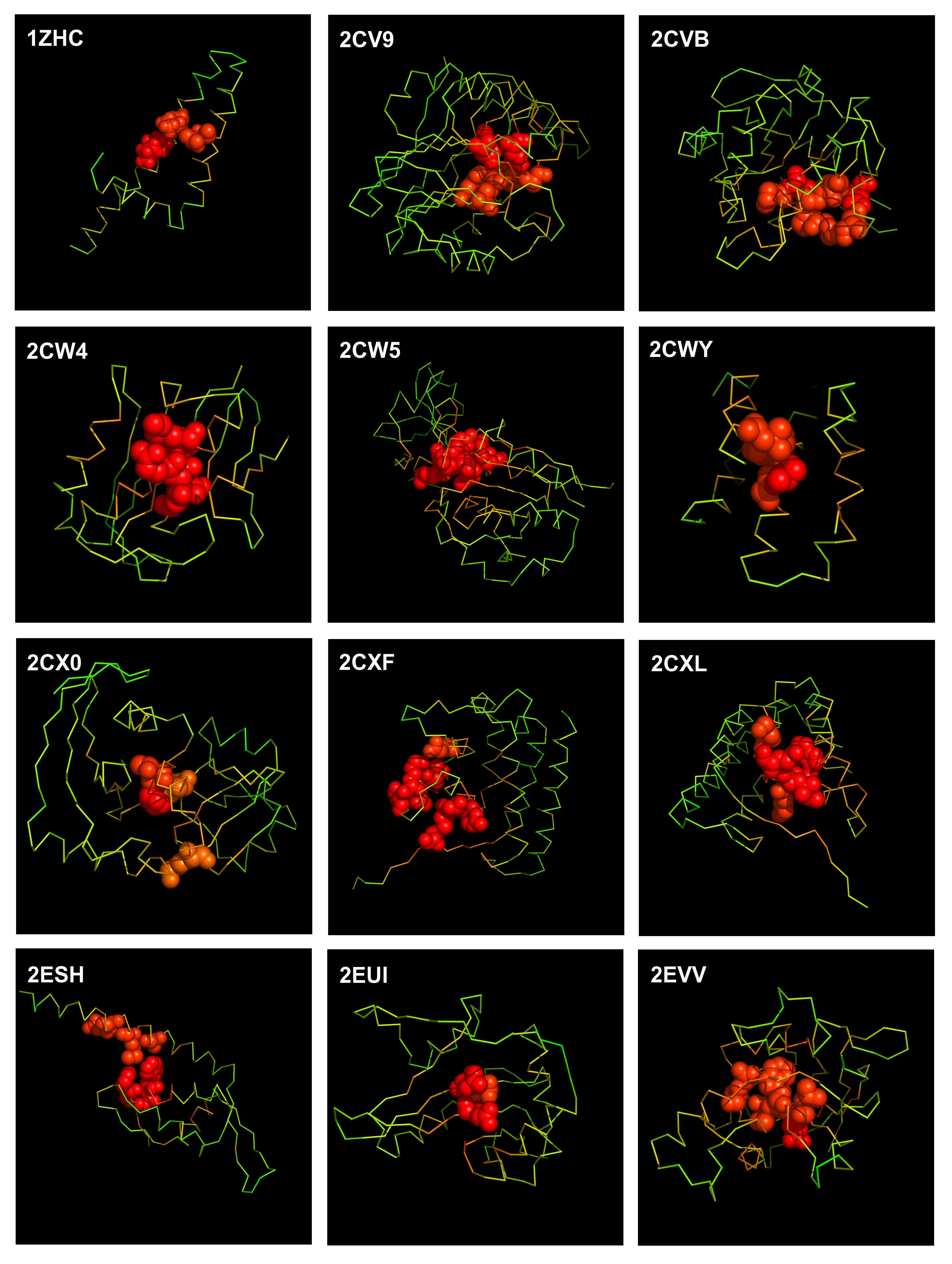

Supplement: Figure S8 — (7.3 MB TIF) [file pcbi.0030094.sg008.tif]

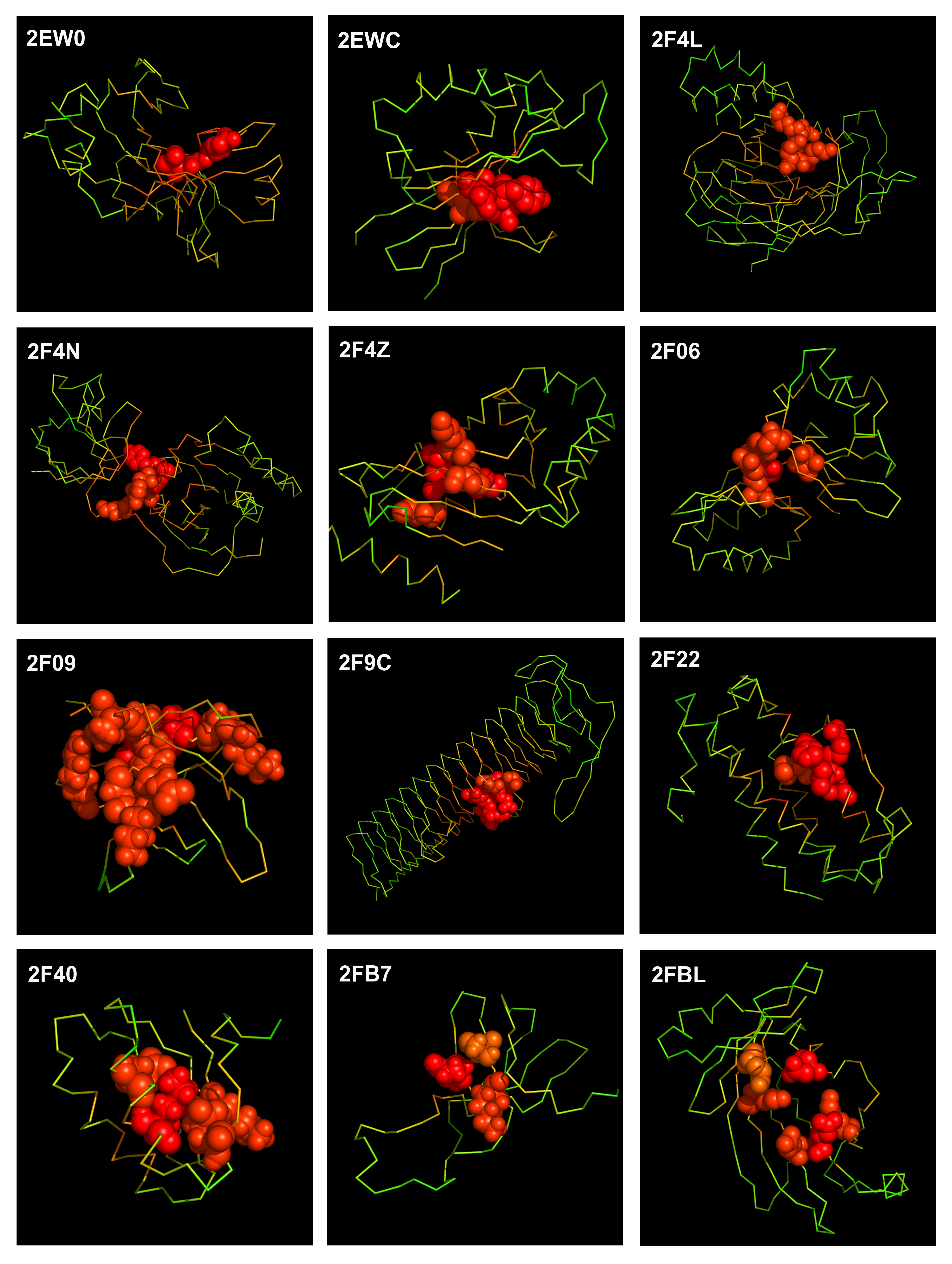

Supplement: Figure S9 — (8.4 MB TIF) [file pcbi.0030094.sg009.tif]

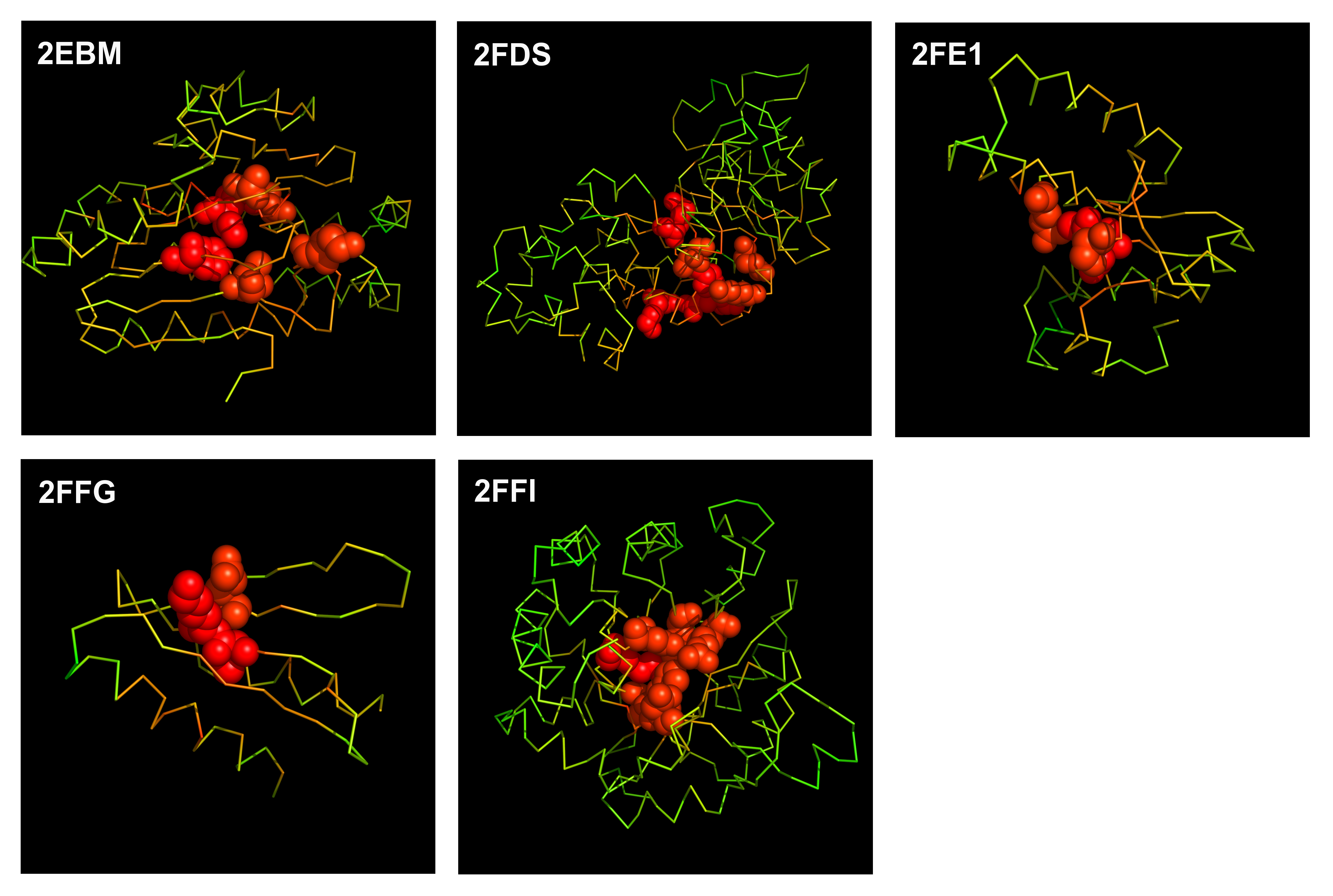

Supplement: Figure S10 — (3.5 MB TIF) [file pcbi.0030094.sg010.tif]
